# Discrimination of temporal fatigue-related states and inferred brain-function coupling in subway drivers under emotional influence

**DOI:** 10.3389/fnhum.2026.1700040

**Published:** 2026-06-05

**Authors:** Jufen Yang, Chenyang Xia, Weikang Wang, Haozhe Yang, Minghui Ma

**Affiliations:** School of Urban Rail Transit, Shanghai University of Engineering Science, Shanghai, China

**Keywords:** CNN-LSTM model, discrimination of temporal fatigue-related levels, Dynamic Bayesian Inference, electroencephalogram, emotional influence, inferred brain-functional coupling, Toeplitz Inverse Covariance-Based Clustering

## Abstract

**Introduction:**

The mental fatigue state of subway drivers directly affects operational safety. At the same time, they are easily influenced by individual emotions and are closely related to the state of brain function.

**Methods:**

In this study, multimodal data of subway drivers during driving were collected synchronously in a simulated driving environment, including EEG signals, subjective fatigue perception, emotional states and operational performance data. First, the Toeplitz inverse covariance clustering method was used to integrate the subjective fatigue perception, emotional states and operational performance data of subway drivers to derive four data-driven fatigue states. Subsequently, the EEG signals were decomposed into four frequency bands of *θ*/*α*/*β*/*γ*, and the time domain, frequency domain and spatial domain feature indicators were extracted as input data. A discrimination model for the four data-driven fatigue states based on a convolutional recurrent neural network was constructed. After multiple sets of feature set combinations and integrated strategy tests, the optimal fatigue levels discrimination model was finally selected. Further, Dynamic Bayesian Inference was used to explore the of inferred brain-functional coupling patterns of the EEG signals corresponding to each data-driven fatigue state.

**Results:**

The results showed that across the four data-driven fatigue states, the prefrontal cortex consistently occupied a central role in task planning and execution, while the other brain regions formed a dynamically coordinated network through bidirectional drive and feedback relations, suggesting systematic differences in inter-regional functional coupling patterns across the identified states.

## 1 Introduction

### 1.1 Research background and motivation

With the improvement of the intelligence level of urban rail transit systems, subway drivers’ tasks have gradually shifted from traditional control to monitoring-based responsibilities, but they still have to face high-strength mental workloads and complex and changing operating environments. Long-term shifts, irregular work and rest schedules, underground closed spaces, and continuous monotonous work make them more susceptible to physical and mental disruption, and fatigue is particularly prominent (Dorrian et al., 2011). Fatigue not only affects the subway driver’s attention maintenance and operational reaction speed, but also poses a risk to the safe operation of urban rail transit (Lal and Craig, 2001). In recent years, more and more studies have found that emotional state is an important factor affecting the formation and evolution of fatigue. Negative emotions not only increase the psychological burden of individuals, but may also trigger the physiological and behavioral manifestations of fatigue in advance (Matthews and Desmond, 2002). Compared with the focus on workload and physiological factors in traditional fatigue research (Hancock and Verwey, 1997), research on emotions as fatigue inducers is still relatively limited. Experimental evidence has shown that when individuals are depressed, anxious, or angry, they are more likely to perceive fatigue and show faster cognitive resource depletion during tasks (Hockey, 2013). This shows that emotions may profoundly affect the occurrence and development of fatigue by regulating arousal levels, resource allocation mechanisms, and other pathways (Russell, 1980). Although relevant studies have initially revealed the impact of emotions on fatigue, in the field of rail transit, there is still a lack of systematic empirical research based on real or highly simulated operational environments. In particular, there is no unified understanding of the mechanism of how emotions affect the evolution of fatigue. At the same time, existing studies have made some progress in revealing the changes in operational performance and subjective perception of fatigue, but there is still insufficient attention paid to the physiological basis of brain function behind the fatigue state (Borghini et al., 2014). Especially in the process of task execution, how the brain’s functional state responds to emotional changes and participates in the dynamic process of fatigue evolution, there is a lack of sufficient empirical evidence (Lal and Craig, 2002). Therefore, it is urgent to integrate multi-dimensional data such as emotions, operational performance and subjective perception in a context that is closer to the actual operation environment, to conduct in-depth exploration of the formation mechanism of fatigue state, and to further reveal its evolution characteristics and regulation patterns from the perspective of brain function (Poldrack et al., 2009).

### 1.2 Current status of research on the correlation between emotion and fatigue

The interaction between driving fatigue and emotional state has become an important issue in the field of traffic safety. Recent studies have shown that emotions significantly regulate the generation and accumulation of fatigue by affecting the driver’s physiological arousal, cognitive resource allocation and behavioral decision-making. Emotions are a core component of human psychological activities, and their complexity is often analyzed through multidimensional models. In the field of psychology, valence, arousal and dominance are widely considered to be the three core dimensions of emotions. The use of the Manikin Self-Assessment Scale to simultaneously measure emotional valence, arousal, and dominance revealed that high negative valence emotions were significantly correlated with self-rated fatigue scores (Bradley and Lang, 1994). Research conducted in a driving simulator environment incorporating the Driver Stress Screening Questionnaire (DSSQ) confirmed that stress-related emotions can predict fatigue levels (Matthews et al., 1998). Through physiological signal detection and heart rate variability (HRV) analysis, which reflects the balance between sympathetic and parasympathetic nervous activity, it was found that negative valence emotions reduce HRV and accelerate fatigue (Healey and Picard, 2005). Sleep deprivation experiments demonstrated that depression combined with fatigue leads to a 35% increase in reaction time (Åkerstedt et al., 2010). Functional magnetic resonance imaging (fMRI) studies indicated that negative emotions activate the amygdala and inhibit the prefrontal cortex (PFC), thereby impairing decision-making control under fatigue (LeDoux, 2013). In the context of deep learning, an approach based on Long Short-Term Memory (LSTM) networks was used to capture progressive fatigue characteristics, it was verified that a shift in emotional valence from positive to negative can provide a 10-min advance warning of fatigue (Wijnands et al., 2018).

### 1.3 Current status of research on fatigue state evolution

The temporal change of fatigue state is a complex process, which is influenced by biological, physiological, psychological, and other factors. It follows a pattern of accumulation over time. If short-term fatigue is not alleviated, it can lead to increased fatigue. To study the temporal change of fatigue, it is necessary to first classify the fatigue level. Traditional studies simply divide the fatigue state into “awake” and “fatigue,” but this binary classification fails to capture the cumulative and transitional nature of fatigue (Williamson et al., 2011). To address this limitation, a four-level fatigue classification system (awake, mild fatigue, severe fatigue, sleepiness) was developed based on electroencephalogram (EEG) signals and modeled using a bidirectional long short-term memory (LSTM) network. This multi-level classification approach proved more effective in capturing temporal characteristics of fatigue, achieving a classification accuracy exceeding 90% (Hu and Lodewijks, 2020). Fatigue level classification primarily relies on trends in physiological and behavioral indicators. Commonly used physiological signals include EEG, HRV, electrooculogram (EOG), and electromyography (EMG) (Jap et al., 2009; Kołodziej et al., 2020; Tran et al., 2009; Dimitrova and Dimitrov, 2003). Among these, EEG signals are widely utilized due to their high sensitivity to cognitive and mental states (Lotte et al., 2018). To enhance the objectivity and scientific rigor of fatigue level classification, several studies have combined subjective fatigue assessment scales with behavioral response tasks. For instance, the Karolinska Sleepiness Scale (KSS) was integrated with the Psychomotor Vigilance Task (PVT) to label fatigue states in a simulated driving experiment, with subsequent physiological validation via EEG measures. The results confirmed a strong correlation between subjectively reported fatigue levels and electrophysiological features derived from EEG (Eoh et al., 2005). Such multimodal labeling approaches provide a reliable foundation for subsequent fatigue detection modeling. Based on well-defined fatigue classification criteria, researchers have developed various models for the automated recognition and continuous monitoring of fatigue states. Traditional methods primarily rely on machine learning techniques such as support vector machines (SVM), k-nearest neighbors (k-NN), and decision trees (DT) (Hasni et al., 2017; Rashid et al., 2021; Shen et al., 2006), these approaches depend on handcrafted features extracted from time-domain and frequency-domain representations of EEG, HRV, and other signals. A driving fatigue discrimination model based on EEG signals was developed using support vector machine (SVM), achieving over 85% accuracy in a three-class classification task. This result demonstrates the feasibility of traditional machine learning methods for static fatigue assessment (Yeo et al., 2009). To better capture temporal dynamics in fatigue detection, deep learning methods have been increasingly employed in recent years. A deep neural network architecture was proposed that integrates EEG signals with cognitive load prediction and uses Long Short-Term Memory (LSTM) modules to model the temporal evolution of fatigue. Validation results demonstrated that this approach can effectively predict changes in fatigue levels and provide early warnings for abnormal states (Zheng and Lu, 2017). Furthermore, with the rise of the Transformer architecture in time-series modeling, some studies have begun exploring its potential for EEG-based fatigue monitoring, showing enhanced capability in capturing long-range dependencies (Li et al., 2024). Additionally, intelligent optimization algorithms have been incorporated to enhance model parameter tuning and feature selection. For instance, particle swarm optimization (PSO) was combined with support vector machine (SVM) to optimize kernel function parameters and feature subsets, thereby improving the accuracy and robustness of fatigue level discrimination (Zeng et al., 2022).

### 1.4 Current status of research on brain function mechanisms

The relationship between brain function and fatigue has become a significant research focus in the fields of neuroscience and cognitive psychology. With continuous advancements in brain imaging technology and EEG signal analysis, a growing number of studies are investigating changes in brain functional mechanisms under fatigue conditions. Research has shown that during fatigue, low-frequency neural oscillations (*θ*/*α*) are significantly enhanced, while high-frequency activity (*β*/*γ*) decreases, suggesting that fatigue may induce a low-efficiency state in the brain, thereby impairing cognitive and decision-making functions (Tran et al., 2020). Furthermore, brain regional functions vary considerably throughout the progression of fatigue (Ishii et al., 2014). The PFC, which is critical for decision-making, planning, attention control, and response inhibition, shows reduced activation as fatigue increases, resulting in impaired attention and poorer judgment. Meanwhile, regions such as the amygdala and hippocampus, which are associated with emotional and memory processing, become more active under emotional fatigue. Activation of the amygdala in particular can intensify negative emotions, thereby exacerbating cognitive fatigue (Yoo et al., 2007). Studies employing functional connectivity analysis have further demonstrated that fatigue significantly alters functional connections among multiple brain regions, especially between the PFC and the amygdala (Dimitrakopoulos et al., 2018). Fatigue impairs the brain’s capacity to regulate emotions and support decision-making, increasing error rates during stressful and complex tasks. As fatigue deepens, functional connectivity across brain networks undergoes dynamic reorganization (Barrett and Satpute, 2013). It has been shown that fatigue not only influences activity in isolated brain regions but also modulates information flow and functional integration across distributed networks. For instance, increased functional connectivity between the PFC and the posterior parietal cortex (PPC) under fatigue may reflect a compensatory mechanism to sustain cognitive performance through enhanced neural communication (Shen et al., 2020). Although substantial research has explored the association between fatigue and brain function, the precise mechanisms underlying fatigue-related changes in brain activity-particularly how emotional states modulate functional networks across different fatigue levels-require further investigation through more refined experimental designs and advanced analytical approaches. Future studies should integrate multimodal neuroimaging techniques (e.g., fMRI and EEG) with computational modeling to unravel the complex interplay between fatigue and brain dynamics, ultimately contributing to more accurate biomarkers for fatigue monitoring and intervention (Michel and Koenig, 2018).

## 2 Problem description

**Figure 1:**
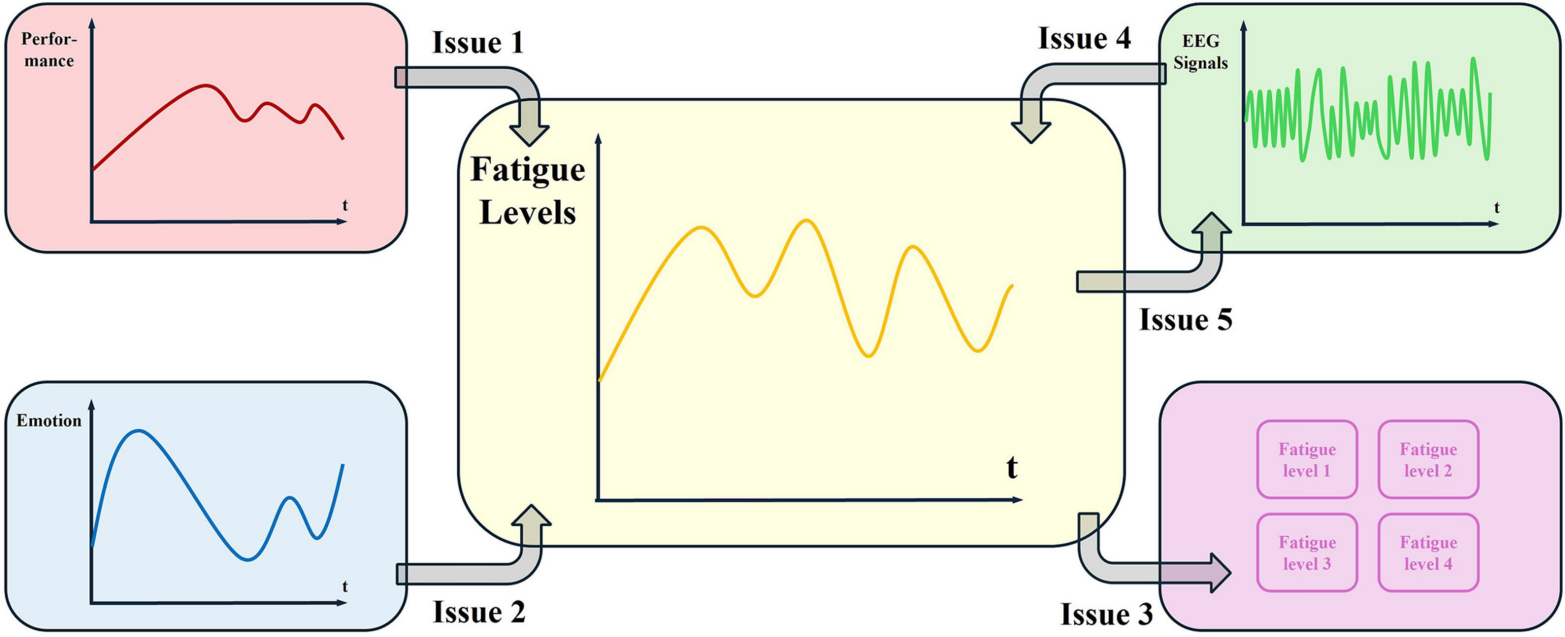
A schematic diagram of the temporal evolution of metro drivers’ operation performance, emotional state, fatigue level, and EEG signals during driving tasks.

The driver’s operational performance, emotional state, fatigue-related level, and EEG signals all exhibit dynamic changes over time as subway driving continues, as shown in Figure 1. The subway driver’s operational performance fluctuates across different periods of duty, which may be influenced by cognitive load and environmental conditions. Meanwhile, the driver’s emotional state is not static during the work process and shows bidirectional variations between positive and negative states. These emotional fluctuations may further influence the accumulation of fatigue through psychological mechanisms. Throughout this process, the driver’s fatigue-related states continue to evolve and demonstrates different degrees of severity. A scientifically-grounded classification of data-driven fatigue states is not only essential for understanding fatigue progression but also a prerequisite for effective monitoring and intervention. Therefore, determining how to appropriately derive and interpret fatigue-related operational states is one of the primary questions of this study. Notably, EEG signals directly reflect brain functional activity and possess predictive value for fatigue status. Conversely, across different data-driven fatigue states, EEG activity patterns across various brain regions are expected to exhibit certain underlying regularities and distinctions. Thus, EEG signals can not only serve as a basis for discrimination of the data-driven fatigue states but also hold potential for revealing inferred brain-functional coupling characteristics. These aspects represent two additional key issues addressed in this study.

In summary, this study will focus on the following key issues:

(1) How is the driver’s operating performance affected by fatigue state (Issue 1)?
(2) How does the driver’s emotional state influence the progression of fatigue (Issue 2)?
(3) How can a scientifically valid and practical classification framework for data-driven fatigue states be established (Issue 3)?
(4) How can the driver’s fatigue-related state be discriminated based on EEG signals (Issue 4)?
(5) What differences exist in inferred brain-functional coupling patterns across different f data-driven fatigue states (Issue 5)?

In this framework, the fatigue categories examined in the present study are not treated as externally predefined labels, but as latent operational states inferred from multimodal temporal patterns. This distinction is important for interpreting the subsequent modeling results: the EEG-based models are designed to discriminate among these data-driven states and to explore their associated brain-functional characteristics within the experimental context, rather than to establish universal diagnostic categories of fatigue.

## 3 Experimental design

In order to accurately replicate and simulate actual working conditions, this study was conducted in a subway simulation laboratory equipped with a simulated driver’s cabin and a mock dispatch room. The simulated cabin is identical to those used in real train operation and is integrated with a simulated line system capable of reproducing all scenarios encountered during actual service. The ambient temperature was maintained at 24.5 °C in accordance with national standards, while lighting conditions mirrored those of actual subway lines. Noise levels were controlled at approximately 85 dB to closely mimic real-world operating conditions.

Recruit 4 personnel from those with internship experience in urban rail transit. All subjects had undergone formal theoretical training and practical operational exposure. They were required to abstain from alcohol and caffeine for 24 h prior to the experiment and to maintain a schedule consistent with that of subway drivers. Before the formal experiment, a training session and a pre-test were conducted to familiarize the participants with the subjective evaluation scales, thereby ensuring accurate real-time self-assessment of their state.

The cognitive task was designed and operational performance data were collected using E-Prime 2.0 software. Based on the actual operational context of Shanghai Metro Line 3, red, yellow, and green signal lights were simulated between stations, with blue added as a cognitive distractor. The task followed a Stroop paradigm: each station triggered a 20-s trial during which words were displayed for a maximum of 2 s. Participants were instructed to respond by pressing keys corresponding to the font color rather than the word meaning. Since verbal stimuli spaced more than 2 min apart do not significantly affect physiological trends, experimenters inquired about the participants’ condition every 5 min and simultaneously recorded KSS and Self-Assessment Manikin (SAM) scores (Schmidt et al., 2017).

**Figure 2:**
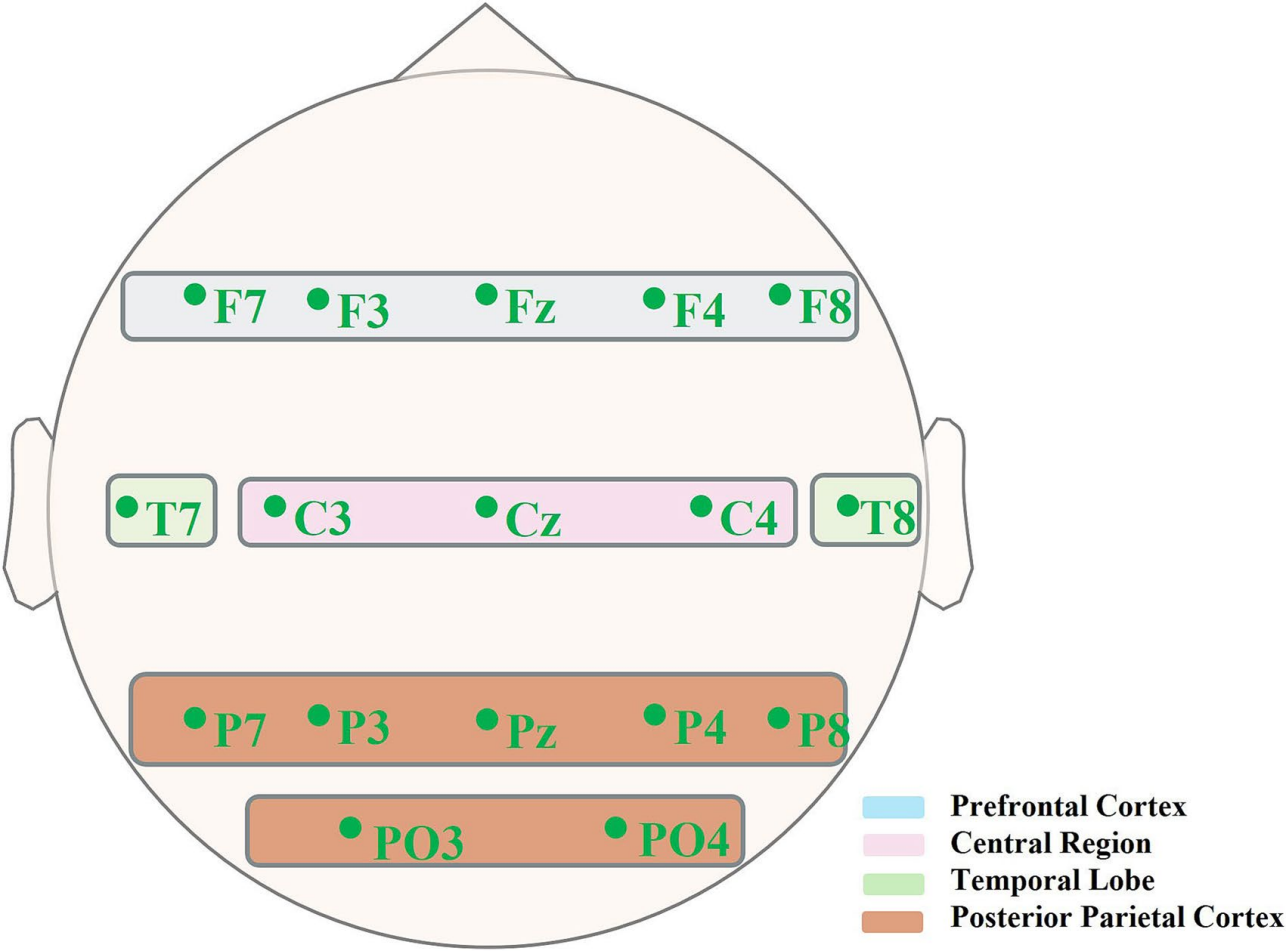
Schematic of electrode layout.

The four types of data collected during the simulated train operation are summarized in Table 1. EEG signals were acquired using a Neuracle 64-channel wireless EEG cap at a sampling rate of 1,000 Hz. The data from the following electrodes were selected for analysis: the PFC (F7, F3, Fz, F4, F8), the central region cortex (CRC) (C3, Cz, C4), the temporal lobe (TL) (T7, T8), and the PPC (P7, P3, Pz, P4, P8, PO3, PO4), as shown in Figure 2. To ensure data quality, the following preprocessing steps were applied: a notch filter was used to remove power-line interference, a FIR bandpass filter (0.1–30 Hz) was applied to attenuate high- and low-frequency artifacts, average re-referencing was employed to reduce common-mode noise, and Independent Component Analysis (ICA) was performed to remove ocular and muscular artifacts. As a sampling rate of 125 Hz has been shown to be sufficient for extracting frequency-domain features related to cognitive fatigue (Lee et al., 2022), the EEG data in this study were downsampled to 200 Hz. This rate retains adequate temporal resolution for time-frequency analysis while reducing computational load. At the end of the experiment, a total of 12,594 s of EEG data were collected, excluding 1,279 s of data due to poor electrode contact, 2,655 s of data severely affected by EEG artifacts, and 860 s of data due to questionnaire and cognitive task errors. The final valid EEG data was 7,800 s.

**Table 1:** Data collection form.

Data type	Specific indicators	Data collection methods
Physiological signals	EEG	Neuracle 64-channel wireless EEG cap
Operational performance data	Reaction time	Eprime2.0
	Accuracy	
Fatigue	fatigue level	KSS scale
Emotion	Valence	SAM scale
	Arousal	
	Dominance	

## 4 Methods

### 4.1 Emotion–fatigue association analysis

In the present study, the relationship between emotion and fatigue is examined primarily from an associative and temporal co-evolution perspective. Because no explicit emotion-induction paradigm, causal intervention, or cross-lagged longitudinal design was implemented, the analyses reported here cannot determine whether emotional changes precede fatigue progression, result from fatigue accumulation, or reflect a bidirectional interaction. Therefore, the findings in this section should be interpreted as evidence of statistical association and temporal coupling, rather than definitive causal direction.

#### 4.1.1 Correlation analysis between emotion and fatigue

Since numerous studies have demonstrated that increasing fatigue significantly impairs drivers’ operational performance, this issue will not be discussed or analyzed further in this study. Pearson correlation analysis was conducted between the subjective KSS scores collected during the test and the three emotional dimensions of the SAM model. The results are presented in Table 2.

**Table 2:** Pearson correlation analysis between KSS and emotional valence, arousal, and dominance.

Index	*F*	*p*
Valence	−0.15	0.056
Arousal	−0.286	<0.01
Dominance	−0.603	<0.01

As shown in Table 2, no significant correlation was observed between emotional valence and KSS scores at the 0.01 significance level (*p* = 0.056). However, both arousal and dominance showed significant negative correlations with KSS, indicating that increased fatigue is accompanied by decreased levels of emotional arousal and dominance. Subjective scale data alone are insufficient to fully elucidate the relationship between fatigue and emotion. Therefore, DE features extracted from EEG signals were incorporated for emotion discrimination. A one-way ANOVA was performed to assess the relationship between DE values across brain regions and emotional valence (Table 3).

**Table 3:** Results of variance analysis of emotional valence DE data in different brain regions.

Position	PFC	CRC	TL	PPC
*p*	0.069	0.201	0.129	0.038

At the 0.05 significance level, changes in subjective emotional valence were found to have a significant effect on DE in the posterior Central Region Cortex. This suggests that DE in this brain region can serve as a useful complement to subjective scales, which alone did not reflect emotional valence significantly. The combined analysis indicates that increased subjective fatigue significantly induces emotional changes, supporting the presence of an interaction between fatigue and emotion.

#### 4.1.2 Temporal co-evolution of emotion and fatigue

**Figure 3:**
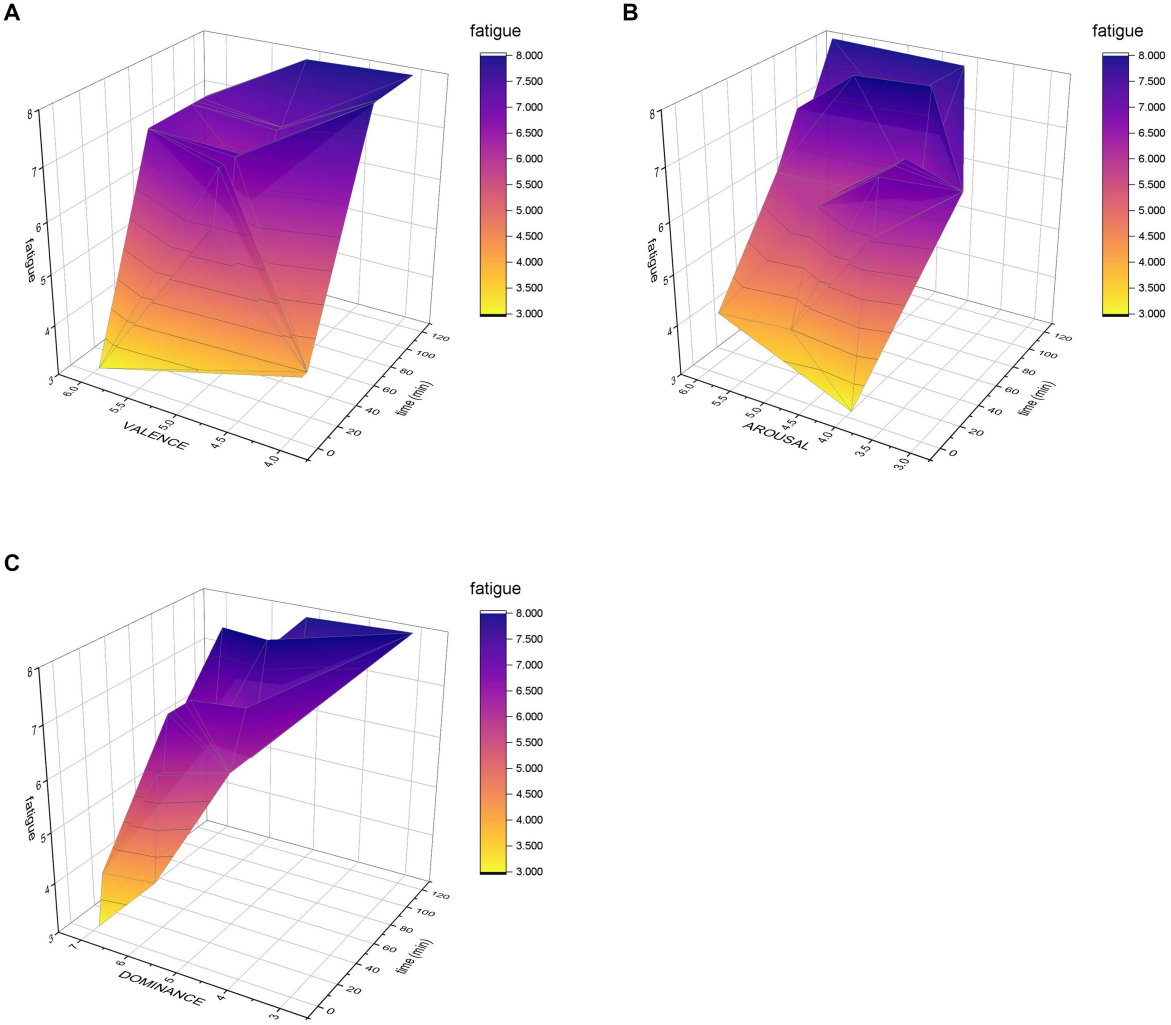
The relationship between fatigue level and the three dimensions of emotion over time: **(A)** Valence; **(B)** Arousal; **(C)** Dominance.

Since the experiment did not employ emotion-induction tasks and instead captured the participants’ natural emotional fluctuations under valid conditions, the observed changes in fatigue under the same experimental setting may be influenced by emotional states. To examine this relationship, a three-dimensional representation of the temporal evolution of emotion and fatigue was constructed, with the three emotional dimensions plotted along the horizontal axis, time along the vertical axis, and fatigue along the depth axis, as shown in Figure 3.

Overall, the temporal trajectories of emotional valence, arousal, and dominance suggest that emotional variation is closely associated with the progression of fatigue during the simulated driving task. In particular, reductions in arousal and more negative emotional tendencies tended to co-occur with higher perceived fatigue. However, given the observational nature of the present design, these results should not be interpreted as establishing a unidirectional causal pathway. Rather, they indicate that emotion and fatigue may evolve jointly and interactively under prolonged operational demands.

### 4.2 Driver fatigue levels classification model based on TICC

Referring to a study on mental fatigue identification through the integration of eye movements, operational performance, and subjective indicators using Toeplitz Inverse Covariance-Based Clustering (TICC) (Li et al., 2020), this experiment integrated input data into a 6-dimensional time series consisting of SAM emotional valence, arousal, dominance, operational reaction time (RT), accuracy (ACC), and KSS scores. According to the TICC method, the input at each time point *t* is a six-dimensional vector (Equation 1). To model temporal dependencies, a sliding window of width *w* is used to concatenate consecutive observations into a high-dimensional vector, forming a subsequence (Equation 2). Here, *w* denotes the window size, and ${\hat{X}}_{t}$ comprises all data from time *t* to *t + w −* 1.


$$X_{t}={\left[\;valence,\;arousal,\;dominance,\;RT,\;ACC,\;KSS\right]}^{T}\in R^{6}$$ (1)

$${\hat{X}}_{t}={\left[X_{t}^{T},\;X_{t+1}^{T},\ldots ,\;X_{t+w-1}^{T}\right]}^{T}\in R^{6w}$$ (2)

The method assumes *k* clusters, each corresponding to a distinct latent operational state related to fatigue progression. Each cluster *k* is modeled as a Gaussian Markov Random Field (MRF) characterized by a precision matrix $\Theta_{k}$ (Equation 3), which follows a block-Toeplitz structure (Equation 4). Each block $A^{\left(d\right)}\in R^{6\times 6}$ encodes conditional dependencies at time lag *d* (e.g., $A^{\left(0\right)}$ represents the relationship between the six dimensions at the same time point; $A^{\left(1\right)}$ represents the relationship between two adjacent time points), thereby capturing both within-time interactions and cross-time dynamic relationships among the variables.


$$p\left({\hat{X}}_{t}|\Theta_{k}\right)\propto \exp\left(-\frac{1}{2}{\hat{X}}_{t}^{T}\Theta_{k}{\hat{X}}_{t}\right)$$ (3)

$$\Theta_{k}=\left[\begin{array}{ccc} A^{\left(0\right)} & {\left(A^{\left(1\right)}\right)}^{T} & \cdots \;\;{\left(A^{\left(W-1\right)}\right)}^{T}\\ A^{\left(1\right)} & A^{\left(0\right)} & \begin{array}{cc} \cdots & {\left(A^{\left(W-2\right)}\right)}^{T} \end{array}\\ \begin{array}{c} \vdots \\ A^{\left(w-1\right)} \end{array} & \begin{array}{c} \vdots \\ A^{\left(w-2\right)} \end{array} & \begin{array}{c} \begin{array}{cc} \ddots & \;\;\vdots \end{array}\;\\ \begin{array}{cc} \cdots & \;\;\;A^{\left(0\right)}\;\; \end{array} \end{array} \end{array}\;\right]$$ (4)

The cluster assignments *P* and precision matrices $\Theta_{k}$ are jointly estimated by solving the optimization problem in Equation 5. This objective function comprises: a sparsity-promoting term on $\Theta_{k}$ to ensure interpretability of the dependency network, a likelihood term encouraging data fidelity to the assigned cluster’s distribution, anda temporal smoothing term that penalizes frequent state transitions, regulated by the hyperparameter *β*.


$$\begin{array}{l} \min_{\Theta ,P}\sum_{k=1}^{K}[\underset{\text{Sparsity}}{\underset{︸}{{\left\|\Theta_{k}\right\|}_{1,\mathrm{off}}}}+\sum_{{\hat{X}}_{t}\in P_{k}}\underset{\log-\text{Likelihood}\;\mathrm{Fit}}{\underset{︸}{\left(-\log\;p({\hat{X}}_{t}|\Theta_{k})\right)}}]\\ +\beta \underset{\text{Temporal Switch Penalty}}{\underset{︸}{\sum_{t}1\{P_{t}\neq P_{t-1}\}}} \end{array}$$ (5)

Based on the clustering outcome, the number of clusters was set to *k* = 4, and the resulting clusters were interpreted as four data-driven fatigue states. These four clusters were interpreted as four data-driven fatigue states according to their multimodal profiles and were provisionally described as State 1 (alert), State 2 (mild-fatigue), State 3 (moderate-fatigue), and State 4 (fatigue-affected). It should be emphasized that these labels do not represent externally validated clinical categories; rather, they serve as operational descriptors of latent states identified from the joint dynamics of subjective fatigue, emotional dimensions, and operational performance. To reduce reliance on prior assumptions, the silhouette coefficient was also used to evaluate clustering validity, and the four-cluster solution showed good agreement with the intrinsic structure of the multimodal data.

### 4.3 Fatigue levels discrimination model based on CNN and LSTM

#### 4.3.1 Feature data combination

The original EEG signals were decomposed into four rhythmic bands: *θ* (4–8 Hz), *α* (8–12 Hz), β (12–30 Hz), and *γ* (30–50 Hz)—using Fourier transform and a Butterworth filter. From these bands, Power Spectral Density (PSD) and Differential Entropy (DE) features were extracted. DE reflects changes in fatigue and emotion through signal complexity, with positive values indicating higher complexity and negative values indicating regularity. It is a widely used feature in EEG-based emotion recognition (Zheng and Lu, 2015). While EEG band features including *δ* (0–4 Hz) have been used to detect fatigue in industrial settings (Zhao et al., 2016), the present study excluded the *δ* band due to its strong association with deep sleep. Given the unique responsibilities of subway drivers—where fatigue-induced drowsiness could compromise public safety (Iber, 2007; Dawson et al., 2014)—only the remaining four bands were retained for further extraction of time-domain, frequency-domain, and nonlinear features. While this exclusion was intended to focus the analysis on operational mental fatigue rather than sleep-state classification, it should be regarded as a task-specific methodological choice rather than a universal rule. Because fatigue-related physiology may also involve drowsiness-like dynamics, the possible contribution of δ-band information warrants additional sensitivity analysis and should be examined in future work.

**Figure 4:**
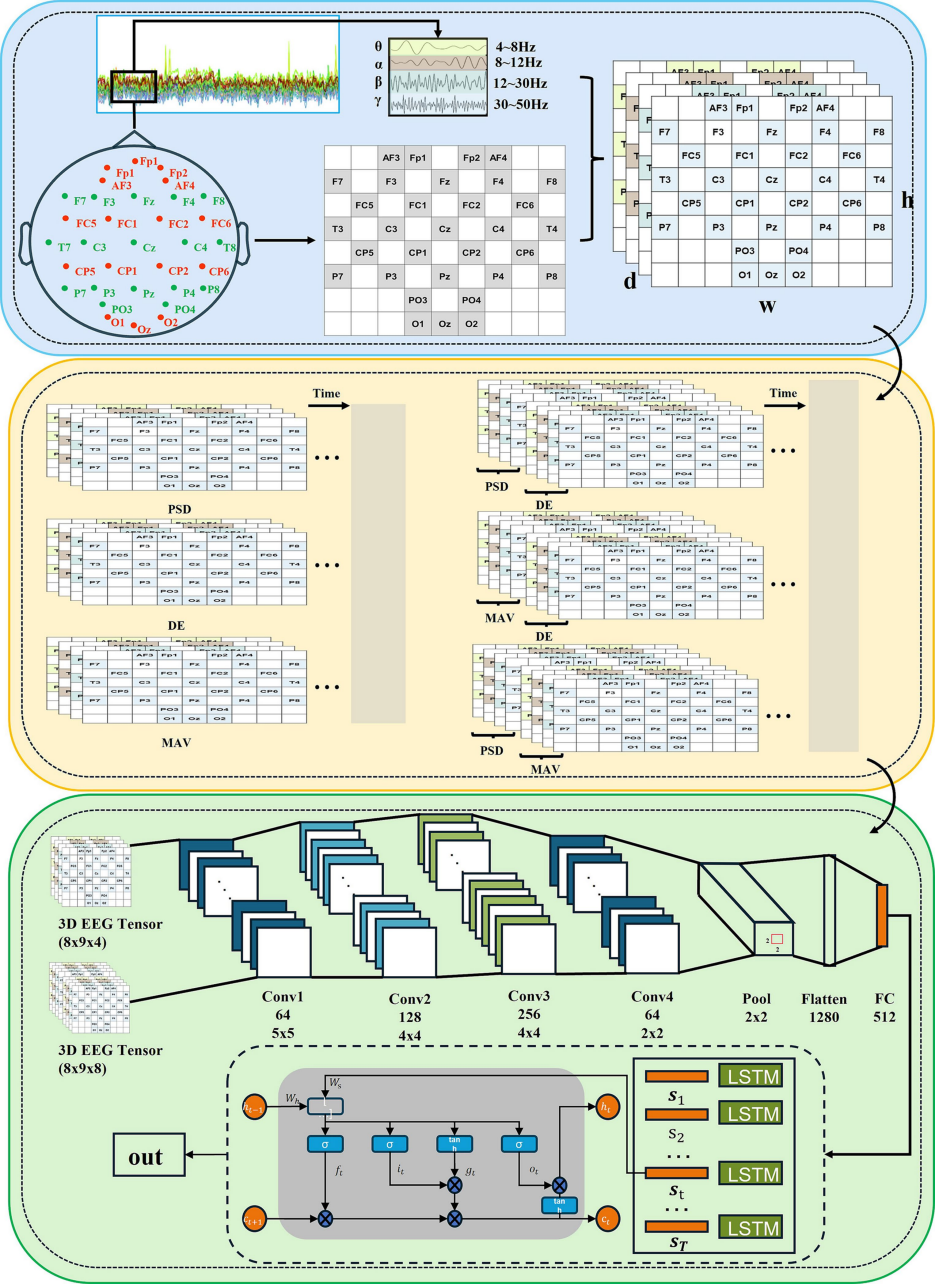
Schematic diagram of the theoretical framework of the CNN-LSTM model.

To integrate time-frequency-spatial information from the 32-channel EEG signals, electrode locations were mapped onto an 8 × 9 two-dimensional brain topography grid (Bashivan et al., 2015). In this grid, only 32 positions contain actual data from the electrodes; the rest are zero-padded to prevent edge loss in the subsequent convolutional processing. Using time-frequency features (Mean Absolute Value (MAV), DE, PSD), the four rhythmic band features were stacked into a three-dimensional structure of size 8 × 9 × 4 within a 10-s time window with 50% overlap. The dimensions correspond to height (h = 8 rows), width (w = 9 columns), and depth (d = 4 frequency bands). Blank electrode positions were filled with zeros to prevent loss of edge information, as shown in Figure 4. To enhance feature complementarity, MAV, DE, and PSD were also combined pairwise to form an 8 × 9 × 8 feature cube (Shen et al., 2020). These datasets incorporate spatial, temporal, and frequency-domain information of the EEG signals, as shown in Figure 4.

#### 4.3.2 Dataset splitting, class distribution, and evaluation setting

Across all valid EEG windows retained after preprocessing, segmentation, and label alignment, the final dataset contained 1,440 samples. For model evaluation, a five-fold cross-validation procedure was adopted under a window-wise data partitioning strategy. In each fold, 80% of the windows (*n* = 1,152) were used for training and the remaining 20% (*n* = 288) were used for testing.

Because the EEG data were segmented using 10-s windows with 50% overlap, adjacent windows were highly correlated, and windows from the same participant could be assigned to different folds. Therefore, the reported classification results should be interpreted as findings obtained under a window-level evaluation setting rather than as definitive evidence of subject-independent generalization.

The final dataset contained 384, 553, 272, and 231 windows for the four data-driven fatigue states (States 1–4), respectively, indicating that the class distribution was not perfectly balanced. To reduce the potential bias toward majority classes, class-weighted cross-entropy loss was applied during model training, with weights assigned inversely proportional to class frequencies. In addition to overall accuracy, Macro-Precision, Macro-Recall, and Macro-F1 were reported to provide a more balanced evaluation of multi-class discrimination performance.

#### 4.3.3 CNN–LSTM architecture

A combined Convolutional Neural Network (CNN) and LSTM computational framework was implemented to discriminate among the four data-driven fatigue states. The overall architecture is illustrated in Figure 4.

The CNN model consists of four convolutional layers, followed by one dropout layer, one batch normalization layer, one max-pooling layer, one flattening layer, and one fully connected layer. The first convolutional layer uses 64 filters of size 5 × 5 to extract preliminary features. This is followed by dropout and batch normalization layers to accelerate training and enhance model robustness. Subsequent convolutional layers gradually capture higher-level features. The resulting features are then processed through a max-pooling layer, flattened into a vector, and fed into a fully connected layer comprising 512 units, which outputs feature time-series data. An LSTM layer with 128 hidden units is then used to process the temporal sequences generated by the CNN, capturing dependencies over time. The LSTM mechanism regulates information flow via input, forget, cell state, and output gates. The final output features are used for discrimination among the four data-driven fatigue states. To prevent overfitting, early stopping was applied by monitoring validation performance; training was halted if the loss showed no improvement over 30 consecutive epochs. The model uses cross-entropy loss to evaluate the difference between predictions and true labels. Optimization algorithms including Adam, SGD, and Adamax were used to update weights and biases, improving convergence speed and overall performance (Lawhern et al., 2018).

#### 4.3.4 Ensemble strategy and training procedure

To enhance the accuracy of multi-class discrimination of the data-driven fatigue states and evaluate the impact of different feature combinations on model performance, this study adopts an ensemble learning strategy to leverage the complementary advantages of multiple base classifiers. Diverse base classifiers were constructed through variations in feature inputs, datasets, or algorithmic structures. The fusion of such heterogeneous models has been demonstrated to improve both robustness and accuracy (Sagi and Rokach, 2018). This study proposes the following three ensemble strategies:

(1) Ensemble of three models using MAV, DE, and PSD as respective feature inputs;
(2) Ensemble of two models selected from the three single-feature models;
(3) Ensemble of two models: one using stacked multi-feature input and the other using one single feature.

The outputs of these ensemble strategies will be integrated using an Unweighted Averaging Method (UWA), a Dynamically Modify Weighting Method (DMW), and a Temporal Voting Algorithm Method (TVA) to determine the final classification result.

### 4.4 Study on the brain function mechanism of subway drivers’ fatigue state

The analyses in this section are intended to characterize fatigue-related changes in inferred brain-functional coupling patterns based on EEG signals. These results should be interpreted cautiously, because effective connectivity estimation is sensitive to preprocessing choices, assumptions of phase-oscillator modeling, and the temporal stationarity of the analyzed signals. Therefore, the DBI-based findings reported here are better understood as model-based evidence of inter-regional neural reorganization under different fatigue states, rather than direct proof of underlying neurophysiological mechanisms.

#### 4.4.1 Effects of fatigue state on brain function

Fatigue and brain function exhibit a complex interplay, directly influencing cognitive processing, perception, and decision-making through inferred coupling patterns between brain regions. Under conditions of anxiety and stress, amygdala activity increases, leading to heightened emotional reactivity, while PFC function—critical for executive control—becomes impaired, resulting in reduced decision-making capacity under pressure. Prolonged exposure to negative emotions and stress may cause overactivation of the central nervous system and even trigger chronic stress responses, further disrupting normal cognitive functioning (Boksem and Tops, 2008).

Under fatigue, PFC-related brain-functional coupling is significantly diminished. This region plays a key role in attention and executive control; as a result, task performance efficiency declines markedly, manifesting as inattention and prolonged reaction times. Furthermore, EEG signals show a pronounced increase in low-frequency *θ*-wave activity, reflecting a shift of the brain into a less efficient mode of operation. These changes indicate that fatigue affects not only subjective experience but also has direct physiological effects on neural function. Positive emotions—such as relaxation and pleasure—play a beneficial regulatory role in brain function. During pleasant states, the brain’s reward system is activated, involving dopamine release that enhances learning and memory. Through the modulation of such neurotransmitters, positive emotions can improve cognitive performance and help restore neural homeostasis.

#### 4.4.2 Analysis of coupling effects of brain function

Interactions between cortical regions of the brain can be quantitatively assessed through effective connectivity methods. Effective connectivity is a mathematical framework within causal modeling that examines how EEG signals influence one another, making it particularly suitable for analyzing synergistic effects of inferred brain-functional coupling across different frequency bands in physiological systems (Bastos and Schoffelen, 2016). Similarly, functional connectivity offers a complementary approach for studying intercortical interactions. Through coupling functions, it becomes possible to elucidate how dynamic neural functions emerge from underlying structural connections.

During task performance, workers process external stimuli that activate or inhibit corresponding cortical regions, leading to correlated EEG signals between these regions. Time-frequency analysis and the construction of coupling functions can help reveal the mechanisms underlying the progression of fatigue. Effective connectivity analysis aids in identifying synergistic frequency bands in EEG, thereby informing subsequent research on neural state interactions. Furthermore, interaction effects can be evaluated using methods such as covariance, spectral coherence, or phase coherence analysis to assess the involvement of various cortical regions under different levels of fatigue.

#### 4.4.3 Introduction to Dynamic Bayesian methods

Dynamic Bayesian Inference (DBI) is a statistical method for analyzing time-series data. It performs effective connectivity analysis on noisy signals with high accuracy and robustness. DBI is particularly valuable because it provides reliable inference results even in the presence of noise or missing data, thereby effectively uncovering the coupling relationships between systems (Stankovski et al., 2015). In the DBI framework, the EEG signal is first transformed into the frequency domain to extract its amplitude and phase, as shown in Equation 6, where A(f) represents the amplitude and φ(f) represents the phase. The phase is derived from the complex angle and is then averaged within the target frequency band to form a phase time series, $\theta_{i}\left(t\right)$ , for each channel, this series serves as the input for subsequent modeling.


$$X\left(f\right)=A\left(f\right)e^{i\varphi \left(f\right)}$$ (6)

Each brain region is treated as a phase oscillator. The dynamics of the coupled phases, which incorporate noise, as shown in Equation 7, where $\omega_{i}$ is the natural frequency, $Q_{ij}$ is the coupling function from oscillator *j* to oscillator *i*, $\Theta \left(t\right)=\left(\theta_{1}\left(t\right),\;\theta_{2}\left(t\right),\ldots ,\;\theta_{N}\left(t\right)\right)$ represents the phase states of all oscillators at time *t*, and $\xi_{i}\left(t\right)$ represents white noise. Equation 7 identifies the coupling functions $Q_{ij}$ and the noise statistics as the key parameters to be estimated. To facilitate estimation, the coupling function $Q_{ij}$ is expanded into a third-order Fourier series, as shown in Equation 8. This parameterizes the coupling relationship into a set of coefficients $\left\{a_{ij,k},\;\;b_{ij,k}\right\}$ , denoted collectively by the parameter β .


$$\frac{d\theta_{i}}{dt}=\omega_{i}+\overset{N}{\underset{j=1}{\sum }}Q_{ij}\left(\Theta \left(t\right)\right)+\xi_{i}\left(t\right)$$ (7)

$$Q_{ij}\left(\Theta \left(t\right)\right)\approx \overset{3}{\underset{k=1}{\sum }}\left\{\begin{array}{l} a_{ij.k}\sin\left(k\left(\theta_{j}\left(t\right)-\theta_{i}\left(t\right)\right)\;\right)\\ +\;b_{ij.k}\cos\left(k\left(\theta_{j}\left(t\right)-\theta_{i}\left(t\right)\right)\;\right) \end{array}\right\}$$ (8)

Equation 7 is then discretized, Denoting the phase difference as Δθ(t)=θ(t+Δt)−θ(t) , the discretized form is given by Equation 9, where *L* is the Cholesky decomposition of the noise covariance matrix. This formulation yields a Gaussian likelihood function. The negative log-likelihood is expressed in a form suitable for recursive updating, yielding estimates for the parameters *β* and the noise statistics.


$$\Delta \theta \left(t\right)\approx \Delta t\left[\omega +Q_{\beta }\left(\Theta \left(t\right)\right)\right]+L\varepsilon_{t},\;\;\varepsilon_{t}\sim N\left(0,1\right)$$ (9)

After estimating β , the coupling strength and direction are defined as shown in Equations 10, 11, respectively. The coupling strength is measured by the Euclidean norm of the Fourier components for the two oscillators, ranging from [0,1] , where a larger value indicates a stronger coupling and the coupling direction is characterized by the difference in these strengths.


$$S_{j\to i}=||{\mathrm{FourierComp}}_{i,j}||\in \left[0,1\right]$$ (10)

$$D_{ij}=S_{j\to i}-S_{i\to j},D_{ij}>0\Rightarrow j\to i$$ (11)

## 5 Results

### 5.1 Model classification performance analysis

Following automated annotation of the multi-source data using the TICC algorithm, the 3D brain maps were input into the CNN-LSTM model constructed in Section 4.3.3 for training. Compared to manual feature annotation, this approach offers improved robustness against signal noise. After five-fold cross-validation and repeated tuning, the Adam optimization algorithm was determined to yield the highest accuracy under both single-feature and combined-feature models. The final model parameters are summarized in Table 4.

**Table 4:** Model parameters.

Parameters	Value	
	Single feature	Multiple features
Initial learning rate	0.001	0.001
Early stopping	True	True
Patience	3 epochs	5 epochs
Dropout rate	0.2	0.6
Batch size	128 sample	256 sample
Epochs	300	500

To evaluate classifier performance, accuracy, precision, recall, and F_1_ score were calculated. Accuracy reflects the overall classification performance, while the F_1_ score represents the harmonic mean of precision and recall. Precision denotes the proportion of true positives among samples predicted as positive, with Macro-Precision being the average precision across all classes. Recall indicates the proportion of true positives correctly identified among all actual positives, and Macro-Recall is the average recall across classes. Macro-F_1_ combines the benefits of both Macro-Precision and Macro-Recall. Table 5 presents the performance metrics under different feature combinations. The data shown in the table are based on a balanced dataset, which was achieved by applying sample balancing techniques to address class imbalance. This balancing was performed prior to the model training process.

**Table 5:** Performance indicators under different feature combinations.

Performance indicators	PSD	DE	MAV	PSD&DE	PSD&MAV	DE&MAV
Accuracy	81.58	88.61	91.06	85.77	91.26	90.49
Macro-precision	79.63	89.57	87.40	87.26	88.02	89.39
Macro-recall	90.56	96.73	95.33	94.63	94.23	98.56
Macro-F_1_	85.18	93.60	92.44	91.57	90.03	95.24

To assess the impact of feature combinations on discrimination of the data-driven fatigue states, model performance was compared between single-feature and multi-feature inputs. The average accuracy of multi-feature inputs was 2.09% higher than that of single features, though not all combinations outperformed single-feature models. For instance, the combination of PSD and DE features achieved higher accuracy than PSD alone but lower than DE alone; similarly, DE combined with MAV outperformed DE alone but underperformed compared to MAV alone. The PSD + MAV combination exceeded the performance of both individual features, indicating that multi-feature integration can enhance performance in certain cases. When using PSD alone, both Macro-Precision and Macro-Recall were relatively low. Other feature inputs achieved an average Macro-Precision of 86.88% and Macro-Recall of 95.01%. Multi-feature inputs improved average Macro-Recall by only 1.60% compared to single features. The DE + MAV combination achieved the highest Macro-F_1_ score (95.24%), indicating the most balanced performance across all four data-driven fatigue states.

**Figure 5:**
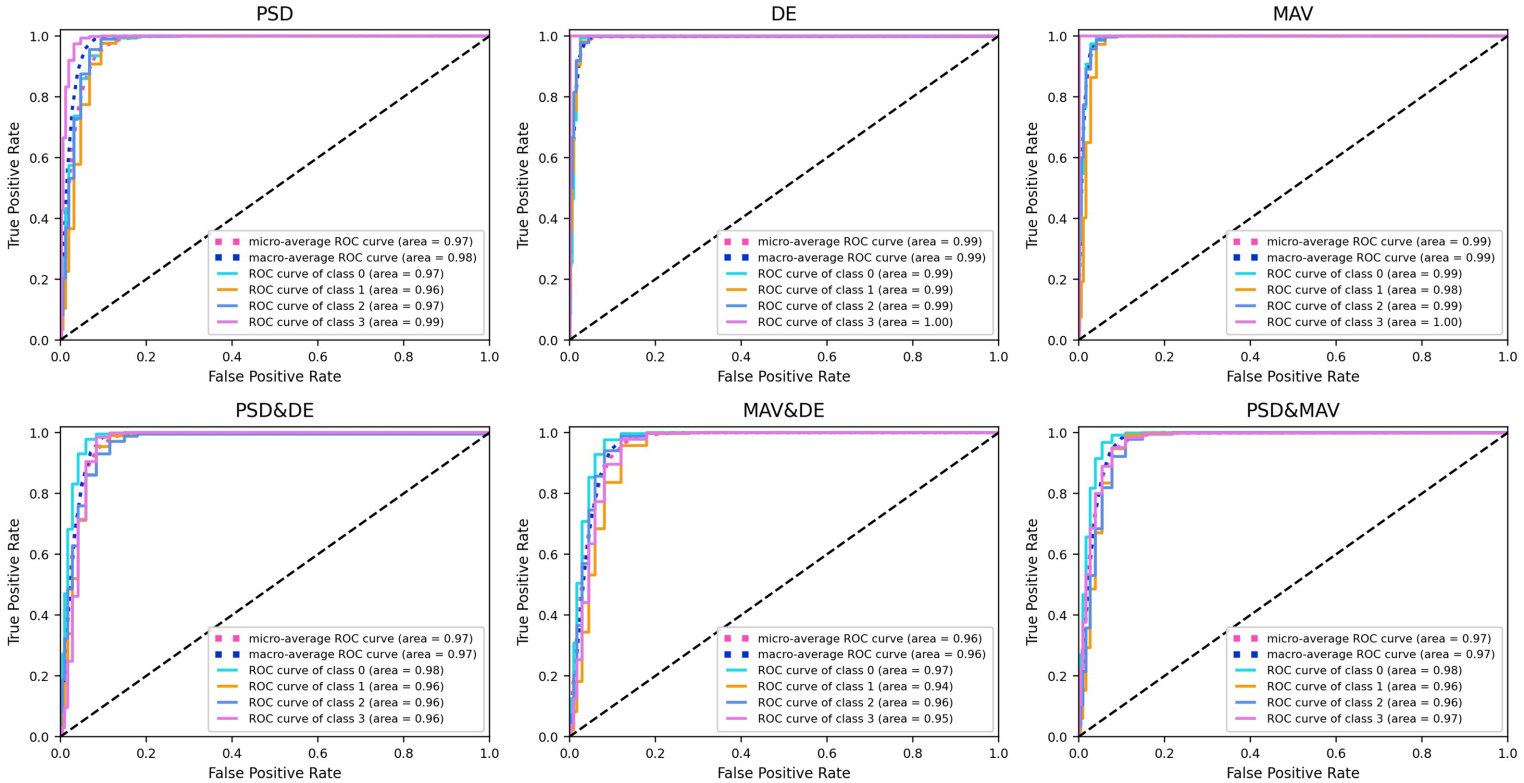
ROC curves of four classifications of six models.

The ROC curve, which illustrates the relationship between the true positive rate and false positive rate, was plotted for all six models (Figure 5). The area under the ROC curve (AUC) was used to evaluate classification performance, with higher values indicating better predictive ability. The average micro-AUC was 97.50%, and the average macro-AUC was 97.67%. Single-feature models generally outperformed multi-feature models in terms of AUC. The DE + MAV combined model achieved the best performance in accuracy, Macro-Recall, and Macro-F_1_, making it suitable for applications where computational cost is not a primary constraint. In contrast, the MAV single-feature model offered the highest accuracy and high Macro-Recall, rendering it appropriate for relatively rapid training and identification of higher fatigue-related levels. In summary, the DE + MAV multi-feature input provides the best overall discrimination of the data-driven fatigue states, while the MAV single-feature input offers an efficient alternative when computational efficiency and detection of higher fatigue-related states are prioritized.

For classification tasks, it is often desirable to improve the accuracy of certain models. Table 6 presents the results of the ensemble strategies (The symbol * denotes the ensemble of two single-feature models; the symbol & denotes the ensemble of a multi-feature model with a single-feature model). The results demonstrate that ensemble learning effectively enhanced model performance. Strategy 1, Strategy 2, and Strategy 3 improved average accuracy by 2.50, 3.24, and 3.12%, respectively. The UWA, DMW, and TVA improved accuracy by 2.28, 3.30, and 3.60%, respectively. Overall, the ensemble strategies increased accuracy by an average of 3.03%. The TVA applied to base classifiers using DE and MAV single-feature inputs achieved the best performance (ACC = 95.44%), outperforming the original base classifiers by 0.07, 1.23, and 3.55%, respectively. The TVA proved particularly effective, likely due to its noise-robust smoothing characteristics. By integrating base classifiers trained on different features, the ensemble model compensates for the limitations of individual feature combinations and better captures underlying patterns across feature types.

**Table 6:** Results of 3 ensemble strategies [Left (w = 10, overlap = 50%); Right (w = 10, overlap = 0%)].

Integration strategies	Methods		Accuracy (%)	Macro-precision (%)	Macro-recall (%)	Macro-F1
Strategy 1	UWA PSD*DE*MAV		90.35 (87.16)	88.09 (80.31)	96.92 (91.87)	93.84 (86.62)
	DMW PSD*DE*MAV		91.41 (88.44)	92.47 (83.02)	98.21 (92.56)	96.73 (87.21)
	TVA PSD*DE*MAV		90.10 (86.15)	91.12 (84.05)	96.74 (92.01)	95.88 (88.57)
Strategy 2	UWA	PSD*DE	86.67 (84.35)	90.36 (81.05)	96.01 (90.62)	94.96 (85.43)
		PSD*MAV	92.54 (89.14)	91.58 (83.44)	97.88 (92.96)	96.41 (89.12)
		DE*MAV	91.38 (88.34)	87.02 (77.66)	97.43 (91.67)	93.66 (84.35)
	DMW	PSD*DE	87.73 (83.96)	86.41 (79.11)	97.02 (91.93)	93.12 (85.78)
		PSD*MAV	94.77 (92.20)	95.87* (86.12)*	99.34 (94.15)	98.53* (89.47)
		DE*MAV	91.42 (88.33)	93.12 (85.71)	98.36 (93.12)	97.94 (91.06)*
	TVA	PSD*DE	88.53 (85.25)	88.56 (78.97)	95.59 (90.16)	94.78 (85.62)
		PSD*MAV	94.33 (90.71)	93.46 (85.64)	99.72* (95.23)*	98.12 (90.54)
		DE*MAV	95.44* (92.91)*	95.18 (85.80)	97.56 (91.68)	97.86 (88.37)
Strategy 3	UWA	PSD&DE*MAV	90.30 (86.28)	90.04 (82.39)	98.28 (94.03)	95.72 (89.21)
		PSD&MAV*DE	94.01 (91.42)	92.31 (82.58)	96.03 (90.41)	95.48 (86.03)
		MAV&DE*PSD	87.34 (84.11)	86.59 (78.83)	93.94 (89.12)	91.76 (84.92)
	DMW	PSD&DE*MAV	93.54 (90.64)	90.18 (80.65)	95.62 (90.01)	94.21 (85.67)
		PSD&MAV*DE	91.76 (88.34)	91.83 (84.18)	98.41 (93.57)	96.38 (89.42)
		MAV&DE*PSD	89.73 (86.84)	86.63 (77.21)	92.67 (87.12)	91.23 (82.11)
	TVA	PSD&DE*MAV	93.37 (89.59)	92.27 (84.52)	95.51 (90.62)	95.32 (88.64)
		PSD&MAV*DE	92.30 (89.78)	92.04 (82.21)	96.82 (91.35)	95.94 (86.72)
		MAV&DE*PSD	88.86 (82.44)	87.31 (82.07)	92.94 (85.27)	91.68 (85.03)

### 5.2 Feature analysis

This study evaluated three EEG signal features—PSD, DE and MAV—for fatigue state classification. Among these, PSD is widely used in fatigue prediction; however, it demonstrated limited effectiveness in multi-classification tasks, likely due to its low temporal resolution and weak correlation structure, making it more suitable for use in combination with other features to provide complementary information. Some previous researchers also believed that DE features are more suitable for EEG-based vehicle fatigue detection than PSD features (Duan et al., 2013). In contrast, DE showed strong performance in multi-class discrimination, particularly when combined with MAV. This combination not only enriches feature information but also significantly enhances model accuracy. MAV captures amplitude fluctuations in time-series data, making it well-suited for discriminating among multiple data-driven fatigue states, while DE effectively represents signal complexity within sliding time windows. Interestingly, the combination of PSD and DE did not yield the expected improvement, possibly due to feature redundancy or overlapping information content. In conclusion, the fusion of DE and MAV proved most effective for multi-class discrimination of the data-driven fatigue states, whereas PSD appears better suited as a supplementary feature rather than a standalone input.

To verify the stability of the model, we adjusted the size of the time window and re evaluated the accuracy and other properties of the ensemble strategy at w = 10 and overlap = 0. The detailed results are shown in Table 6. The accuracy of the integrated strategy decreased by 2.65–7.22%, which is completely acceptable for us, indicating that our model still has strong robustness.

### 5.3 Analysis of the connection between fatigue state and brain function effect

#### 5.3.1 Analysis of coupling direction between fatigue state and brain function

**Figure 6:**
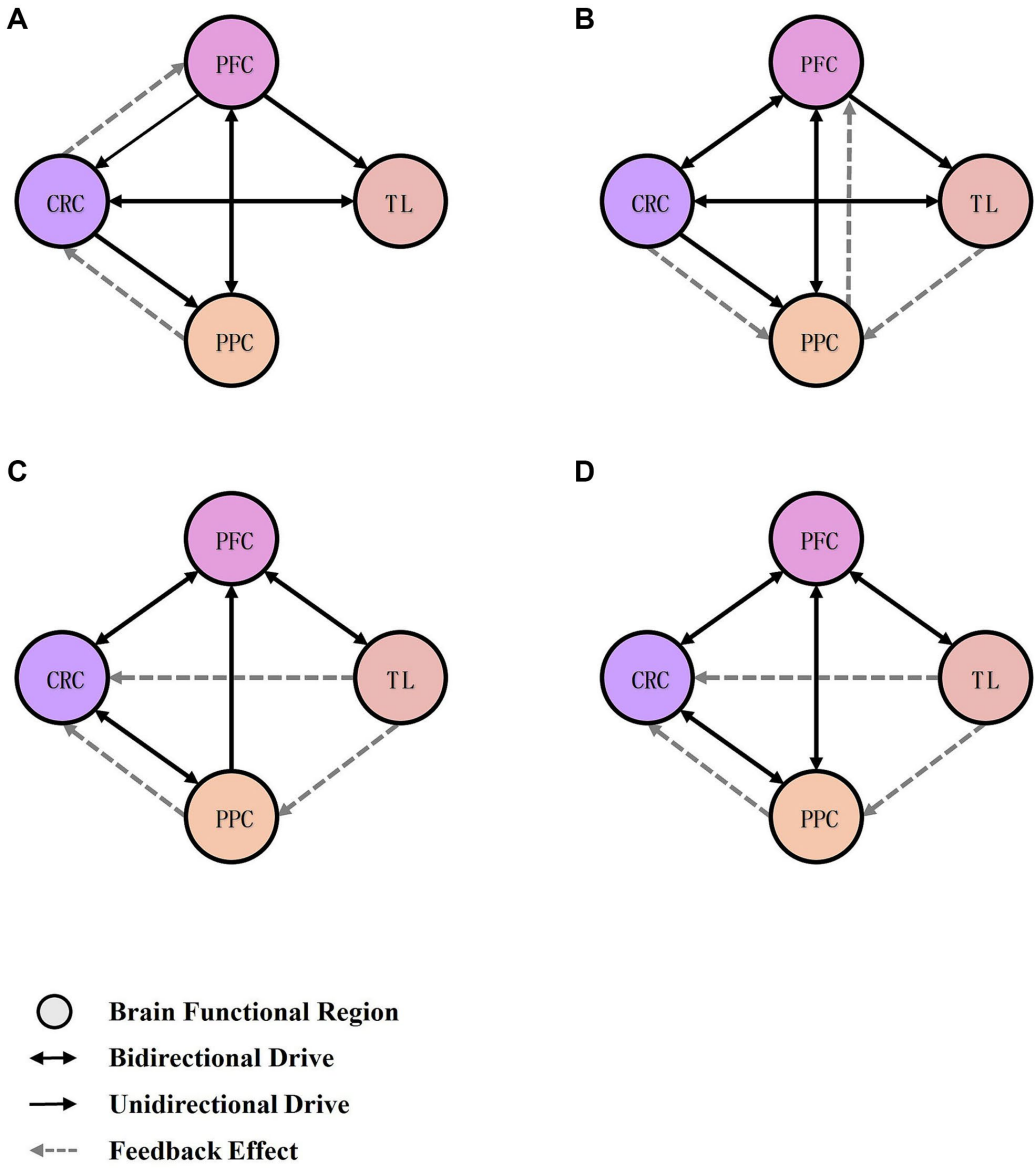
Coupling directions between brain regions across four data-driven fatigue states: **(A)** Alert; **(B)** Mild-fatigue; **(C)** Moderate-fatigue; **(D)** Fatigue-affected.

In studying the evolution of fatigue-related states, the functional influence between different brain regions can be elucidated by analyzing the directionality of inferred brain-functional coupling. By focusing on the directional interactions among major cortical regions and using binomial testing for statistical screening, we summarized the dominant effective-connectivity patterns across the four data-driven fatigue states, including unidirectional drive, bidirectional drive, and feedback relations, as shown in Figure 6. In this framework, unidirectional drive refers to a predominant directional influence from one region to another, bidirectional drive reflects mutual directional interaction, and feedback denotes a recurrent regulatory relation identified in the inferred coupling structure.

In the alert state, the inferred network was characterized mainly by relatively simple directional relations. PFC-to-CRC and CRC-to-PPC interactions were among the more stable direct patterns, suggesting that executive control and sensorimotor coordination were already centrally organized around the PFC-CRC-PPC axis. At this stage, some additional bidirectional or feedback relations were also observed, but their stability was not equally strong across all regional pairs.

In the mild-fatigue state, the inter-regional coupling structure became more complex. Interactions involving PFC-CRC, PFC-PPC, and CRC-TL were more prominent, indicating stronger coordination among executive, perceptual, and task-execution-related regions as fatigue began to accumulate. However, although several feedback relations were present in the inferred network, not all of them were retained with equally high stability across repeated estimations.

In the moderate-fatigue state, the inferred coupling structure showed further reorganization. Interactions involving PPC-to-PFC and CRC-PPC became more prominent, suggesting increased coordination between visuospatial processing and control-execution systems under higher fatigue load. At the same time, recurrent relations involving CRC and TL were also observed, indicating that the brain network may increasingly rely on distributed interaction patterns to maintain task performance under greater cognitive strain.

In the fatigue-affected state, the inferred network remained centered on PFC-related coordination, but the overall interaction pattern appeared more constrained than in earlier stages. Some direct relations persisted, while feedback-related interactions involving CRC and posterior regions remained detectable. Compared with earlier stages, however, several directional relations appeared less stable, suggesting that the network organization under higher fatigue may be more vulnerable and less consistently maintained.

**Figure 7:**
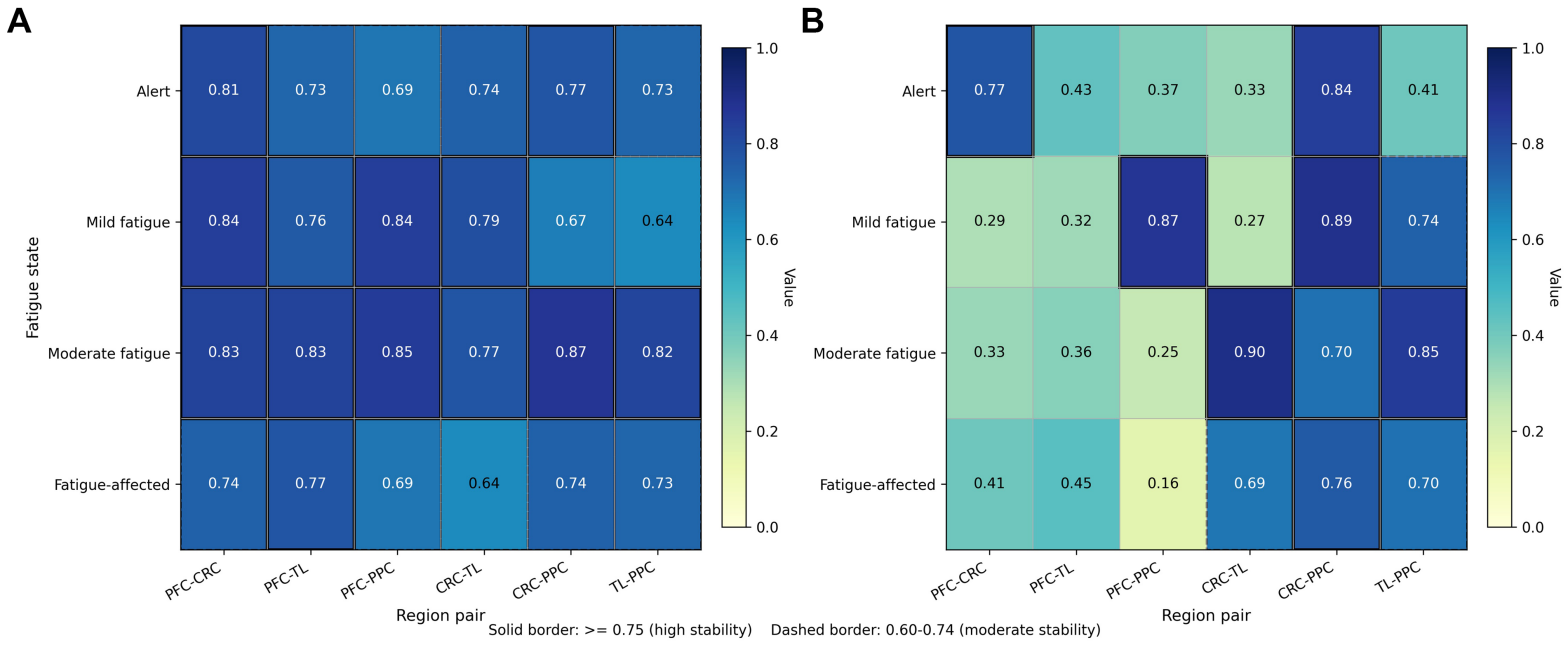
Stability of direct and feedback coupling relationships: **(A)** Direct coupling and **(B)** feedback coupling.

To further evaluate whether the directional relationships shown in Figure 6, were consistently retained across repeated estimations, we summarized the direct proportion and feedback stability of each regional pair across the four fatigue states, as shown in Figure 7. Several core relationships, particularly those involving PFC-CRC and CRC-PPC, showed relatively high stability across states. By contrast, some feedback relations, especially those involving PFC-PPC or PFC-TL in specific states, showed only moderate or limited stability. Therefore, the directional patterns shown in Figure 6 should be interpreted as dominant tendencies in the inferred coupling network rather than equally strong evidence for every individual arrow.

Overall, the results suggest that the inferred coupling structure undergoes dynamic reconfiguration as fatigue progresses, while preserving a PFC-centered coordination pattern. From a functional perspective, this pattern is consistent with the interpretation that executive-control regions remain centrally involved throughout the task, whereas the coordination among control, perceptual, and integrative regions changes with fatigue accumulation.

#### 5.3.2 Analysis of coupling strength between fatigue state and brain function

After identifying the four temporally evolving data-driven fatigue states, the coupling strength between functional brain regions was estimated for each state during task performance, the results are presented in Table 7. Overall, the PFC-CRC coupling remained relatively strong across fatigue states, particularly in the *γ* band, suggesting persistent high-frequency coordination between executive-control and central task-execution regions. Couplings involving PFC-TL also remained relatively evident in selected bands, whereas TL-PPC coupling was generally weaker than the other representative inter-regional pairs.

**Table 7:** Brain function coupling strength table.

Fatigue levels	Brain region\frequency band	*α*	*β*	*θ*	*γ*
Alert	PFC-CRC	0.42 (±0.10)	0.53 (±0.12)	0.31 (±0.08)	0.56 (±0.13)
	PFC-TL	0.37 (±0.08)	0.49 (±0.10)	0.28 (±0.07)	0.52 (±0.11)
	PFC-PPC	0.40 (±0.09)	0.51 (±0.11)	0.30 (±0.08)	0.54 (±0.12)
	CRC-TL	0.36 (±0.07)	0.47 (±0.09)	0.27 (±0.06)	0.50 (±0.10)
	CRC-PPC	0.39 (±0.10)	0.50 (±0.10)	0.29 (±0.07)	0.53 (±0.11)
	TL-PPC	0.35 (±0.08)	0.46 (±0.09)	0.26 (±0.06)	0.49 (±0.10)
Mild-fatigue	PFC-CRC	0.40 (±0.09)	0.51 (±0.11)	0.30 (±0.07)	0.55 (±0.12)
	PFC-TL	0.35 (±0.07)	0.47 (±0.09)	0.27 (±0.06)	0.51 (±0.10)
	PFC-PPC	0.38 (±0.08)	0.49 (±0.10)	0.29 (±0.07)	0.53 (±0.11)
	CRC-TL	0.34 (±0.07)	0.45 (±0.08)	0.26 (±0.06)	0.49 (±0.09)
	CRC-PPC	0.37 (±0.09)	0.48 (±0.09)	0.28 (±0.07)	0.52 (±0.10)
	TL-PPC	0.33 (±0.07)	0.44 (±0.08)	0.25 (±0.06)	0.48 (±0.09)
Moderate-fatigue	PFC-CRC	0.39 (±0.09)	0.50 (±0.11)	0.29 (±0.07)	0.54 (±0.12)
	PFC-TL	0.34 (±0.07)	0.46 (±0.09)	0.26 (±0.06)	0.50 (±0.10)
	PFC-PPC	0.37 (±0.08)	0.48 (±0.10)	0.28 (±0.07)	0.52 (±0.11)
	CRC-TL	0.33 (±0.07)	0.44 (±0.08)	0.25 (±0.06)	0.48 (±0.09)
	CRC-PPC	0.36 (±0.08)	0.47 (±0.09)	0.27 (±0.07)	0.51 (±0.10)
	TL-PPC	0.32 (±0.07)	0.43 (±0.08)	0.24 (±0.06)	0.47 (±0.09)
Fatigue-affected	PFC-CRC	0.38 (±0.08)	0.49 (±0.10)	0.28 (±0.07)	0.53 (±0.12)
	PFC-TL	0.33 (±0.07)	0.45 (±0.09)	0.25 (±0.06)	0.49 (±0.10)
	PFC-PPC	0.36 (±0.08)	0.47 (±0.09)	0.27 (±0.07)	0.51 (±0.11)
	CRC-TL	0.32 (±0.06)	0.43 (±0.08)	0.24 (±0.06)	0.47 (±0.09)
	CRC-PPC	0.35 (±0.08)	0.46 (±0.09)	0.26 (±0.07)	0.50 (±0.10)
	TL-PPC	0.31 (±0.07)	0.42 (±0.08)	0.23 (±0.06)	0.46 (±0.09)

As fatigue progressed from the alert state to the fatigue-affected state, the representative coupling strengths of brain regions generally showed a decreasing trend, reflecting a weakening of inferred brain-functional coupling. This trend was more apparent in selected *γ*- and *θ*-band representative pairs, indicating that the inferred efficiency of inter-regional coordination may decline as fatigue accumulates. By contrast, some lower-frequency patterns appeared comparatively more stable, suggesting that not all functional interactions deteriorated at the same rate across fatigue states. These findings are compatible with the interpretation that fatigue is associated with a gradual reorganization of large-scale neural coordination rather than a uniform reduction of all inter-regional interactions.

**Figure 8:**
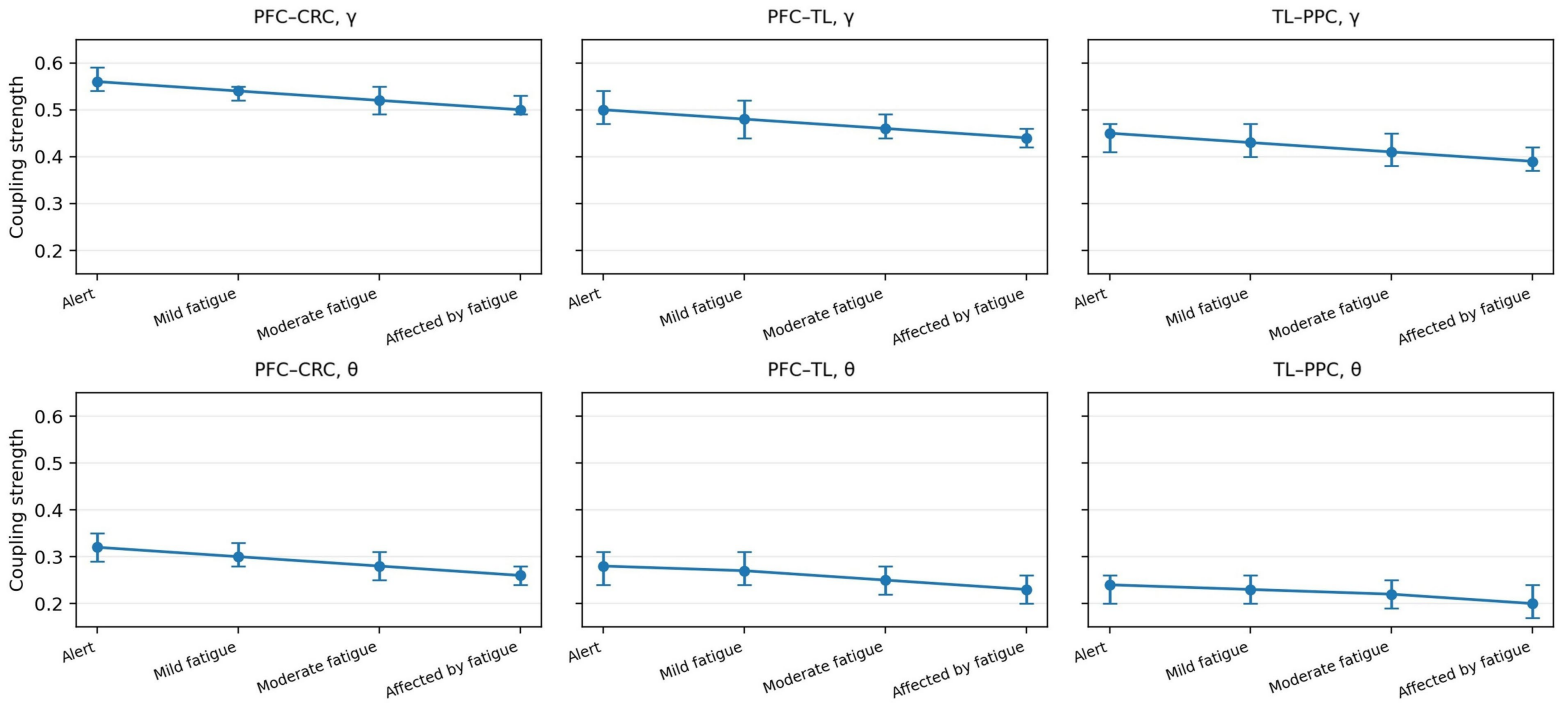
Bootstrapped 95% confidence intervals for representative DBI-derived coupling strengths across the four data-driven fatigue states.

To strengthen the robustness of the DBI-based coupling-strength results, we further calculated bootstrapped 95% confidence intervals for representative inter-regional couplings in the *γ* and *θ* bands, as shown in Figure 8. The selected representative couplings generally exhibited a decreasing trend from the alert state to the fatigue-affected state, and the confidence intervals remained relatively compact at the group level. This suggests that the overall trend was not driven solely by a small number of extreme samples, although the results should still be interpreted as model-based estimates rather than definitive physiological proof.

Taken together, the coupling-strength results indicate that the inter-regional coordination pattern changes systematically with fatigue progression. PFC-related couplings remained among the more prominent interactions across states, whereas the magnitude and stability of several other region-pair interactions declined with increasing fatigue. These findings support the view that fatigue is associated with progressive reorganization of the inferred brain-functional network, while also underscoring the need for cautious interpretation given the assumptions of the DBI framework.

## 6 Future work

Building on the current findings, several avenues will be explored in future research. First, to better understand how emotions influence fatigue progression, Group/Time mixed models or state-space models will be applied to rigorously evaluate the dynamic modulation of fatigue by valence, arousal, and dominance. Second, to more comprehensively capture fatigue mechanisms, additional physiological measures such as heart rate (HR), heart rate variability (HRV), and eye-tracking or oculomotor indices will be integrated alongside EEG. Finally, to enhance the reliability and generalizability of brain-functional coupling findings, cross-participant analyses will be conducted. Approaches including mixed-effects modeling, permutation testing, and inter-subject correlation (ISC) analysis will be used to assess whether observed coupling changes are consistent across individuals, while sensitivity analyses will evaluate robustness to frequency band selection and preprocessing parameters. These efforts aim to strengthen the methodological rigor and translational potential of fatigue monitoring in operational settings.

## Data Availability

The datasets presented in this article are not readily available because these data involve personal privacy issues. Requests to access the datasets should be directed to Chenyang Xia, xiaye2003@126.com.
